# Author Correction: An integrated platinum-nanocarbon electrocatalyst for efficient oxygen reduction

**DOI:** 10.1038/s41467-022-34956-5

**Published:** 2022-11-25

**Authors:** Lei Huang, Min Wei, Ruijuan Qi, Chung-Li Dong, Dai Dang, Cheng-Chieh Yang, Chenfeng Xia, Chao Chen, Shahid Zaman, Fu-Min Li, Bo You, Bao Yu Xia

**Affiliations:** 1 School of Chemistry and Chemical Engineering, Key Laboratory of Material Chemistry for Energy Conversion and Storage (Ministry of Education), Hubei Key Laboratory of Material Chemistry and Service Failure, Hubei Engineering Research Center for Biomaterials and Medical Protective Materials, Wuhan National Laboratory for Optoelectronics, Huazhong University of Science and Technology (HUST), 1037 Luoyu Rd, Wuhan, 430074 China; 2 The Institute for Advanced Studies, Wuhan University, 299 Bayi Rd, Wuhan, 430072 China; 3 Key Laboratory of Polar Materials and Devices (MOE), Department of Electronics, East China Normal University, Shanghai, 200241 China; 4 Department of Physics, Tamkang University, 151 Yingzhuan Road, New Taipei City, 25137 Taiwan China; 5 School of Chemical Engineering and Light Industry, Guangdong University of Technology, Guangzhou, 510006 China

**Keywords:** Catalyst synthesis, Fuel cells, Fuel cells, Materials for energy and catalysis, Nanoscale materials

Correction to: *Nature Communications* 10.1038/s41467-022-34444-w, published online 07 November 2022

The original version of this Article contained an error in Fig. 5d, where the abscissa incorrectly displayed units for the current density of the fuel cell cathodes in mA cm^−2^. This is now corrected and the new figure now displays the units of A cm^−2^ on the abscissa, and does not change the conclusions drawn in the manuscript.

This error has been corrected in both the PDF and HTML versions of the Article.

