# Supplementary material for: An integrated platinum-nanocarbon electrocatalyst for efficient oxygen reduction
Source: Nat Commun. 2022 Nov 7;13:6703. doi: 10.1038/s41467-022-34444-w (PMC9640595; doi:10.1038/s41467-022-34444-w)
Supplement: Supplementary file 1 — Supplementary information [file 41467_2022_34444_MOESM1_ESM.pdf]

Supplementary Information

An Integrated Platinum-Nanocarbon Electrocatalyst for Efficient Oxygen Reduction

Lei Huang [1], *Min Wei* [2], *Ruijuan Qi* [3], *Chung-Li Dong* [4], *Dai Dang* [5], *Cheng-Chieh Yang* [4], *Chenfeng Xia* [1], *Chao Chen* [5], *Shahid Zaman* [1], *Fu-Min Li* [1], *Bo You* [1], and *Bao Yu Xia* [1]*

[1] *School of Chemistry and Chemical Engineering, Key Laboratory of Material Chemistry for Energy Conversion and Storage (Ministry of Education), Hubei Key Laboratory of Material Chemistry and Service Failure, Hubei Engineering Research Center for Biomaterials and Medical Protective Materials, Wuhan National Laboratory for Optoelectronics, Huazhong University of Science and Technology (HUST), 1037 Luoyu Rd, Wuhan 430074, China.*

[2] *The Institute for Advanced Studies, Wuhan University, 299 Bayi Rd, Wuhan 430072, China.*

[3] *Key Laboratory of Polar Materials and Devices (MOE), Department of Electronics, East China Normal University, Shanghai 200241, China.*

[4] *Department of Physics, Tamkang University, 151 Yingzhuan Road, New Taipei City 25137, Taiwan, China.*

[5] *School of Chemical Engineering and Light Industry, Guangdong University of Technology, Guangzhou, 510006, China.*

**Corresponding authors: byxia@hust.edu.cn (B.Y. Xia)*

Supplementary methods

XAS data analysis: Pt foil, PtO_2, Co foil and CoO were used as standard samples to evaluate the coordination environment and chemical state of Pt L_3-edge and Co K-edge of PtCo@CoNC samples. According to the energy correction of Pt foil and Co foil, the signals of Pt L_3-edge and Co K-edge were optimized using the Athena program in the IFEFFIT software package,[1] and then E_0 was determined based on the highest value of the first derivative of XANES. Through the corrected XANES white line peak position, the chemical valence range of Pt and Co in PtCo@CoNC/NTG could be determined. For EXAFS analysis, the k space range (2.0-12.0 $Å^{-1}$) was selected for Fourier transform (FT) to obtain R space, where the k-weight was 2. The first shell of R space was selected for inverse Fourier transform to obtain q space, involving Pt L_3-edge of Pt foil (1.1-3.1 Å), PtCo@CoNC/NTG (1.0-3.0 Å), PtO_2 (1.1-2.1 Å), and Co K-edge of Co foil (1.0-3.0 Å), PtCo@CoNC/NTG (1.0-3.3 Å) and CoO (1.1-2.8 Å). In the subsequent EXAFS fitting, the value range of k and R space was the same as the selection in the abovementioned FT and inverse Fourier transform. According to the set coordination number of Pt foil (12) and Co foil (12), the amplitude reduction factor S_0^2 of Pt L_3-edge and Co K-edge was obtained by fitting the Artemis program. After EXAFS fitting, the detailed fitting information of PtCo@CoNC/NTG could be recorded, including the coordination number, bonding length, Debye-Waller factor, and energy shift., etc. More EXAFS fitting information is shown in Supplementary Table 1 and Table 2.

DFT calculation details: Density-functional theory (DFT) containing spin polarization was implemented in the Vienna *ab initio* simulation package (VASP).[2] The core-electron interactions were described by the projector-augmented wave (PAW) method. The exchange-correlation interaction was represented by the Generalized gradient approximation with Perdew, Bruke and Ernzerhof (PAW-PBE).[3] The plane wave function with cut-off energies of 600 and 400 eV was used for bulk geometry and slab optimizations, respectively. Face-centered cubic structures of metallic Pt and PtCo were used as bulk models. A vacuum thickness of 15

Å was added perpendicular to the surface to avoid the image force between the periodic slab. The convergence criteria of energy and force were set to 1 x 10^{-5} eV and -0.02 eV·$Å^{-1}$ in all calculation processes, respectively. A k-point sampling 5 × 9 × 1 based on Gamma-centred Monkhorst-Pack was conducted for Pt-111 geometry calculation and slab structure relaxations. A four-layer slab model with a supercell (2 × 2) was built by cleaving the (111) facet of metallic Pt. The adsorption energy was calculated according to the formula ΔE_{ads} (O*) $= E_{slab+O}$ (O*) - E_{slab} - 1/2E (O_2), where E_{slab+O} (O*), E_{slab}, and E (O_2) represent the total energy of O adsorption on the slab, the total energy of the pure slab and the total energy of the oxygen molecules in the gas phase, respectively.[4] The Gibbs free energy of the intermediate at 300 K and 1 atm was calculated by $\Delta G = \Delta E_{abs} + \Delta ZPE - T\Delta S$, where ΔE_{abs}, ΔZPE, and ΔS are the adsorption energy, zero-point energy change and entropy change of the adsorption of intermediates, respectively.

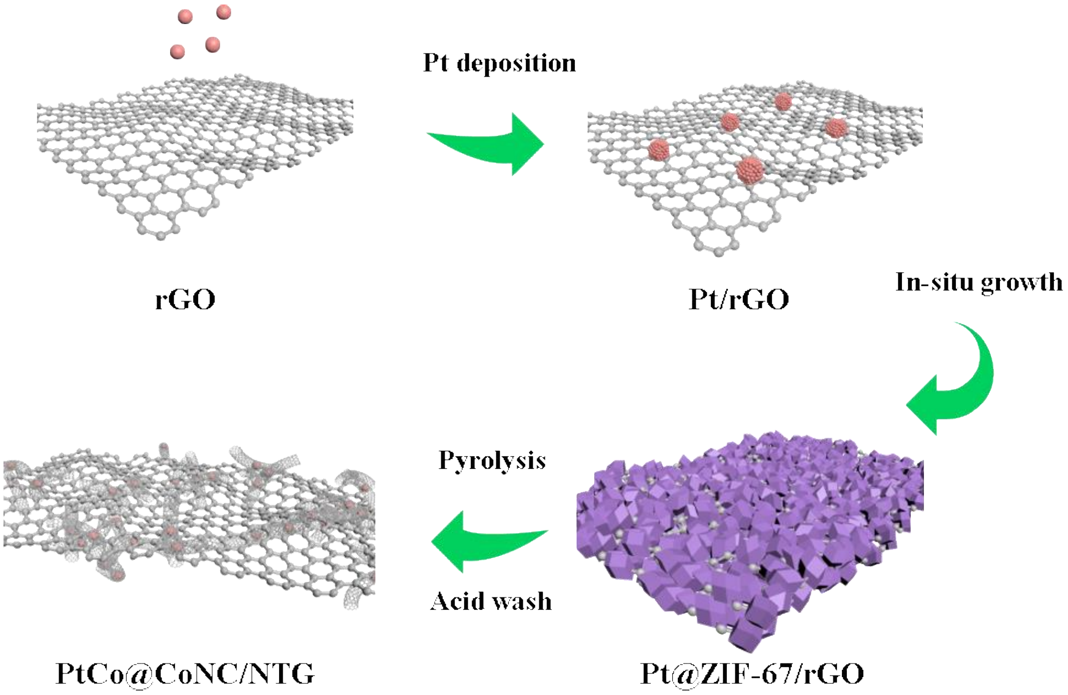

Supplementary Fig. 1 Synthetic illustration of PtCo@CoNC/NTG.

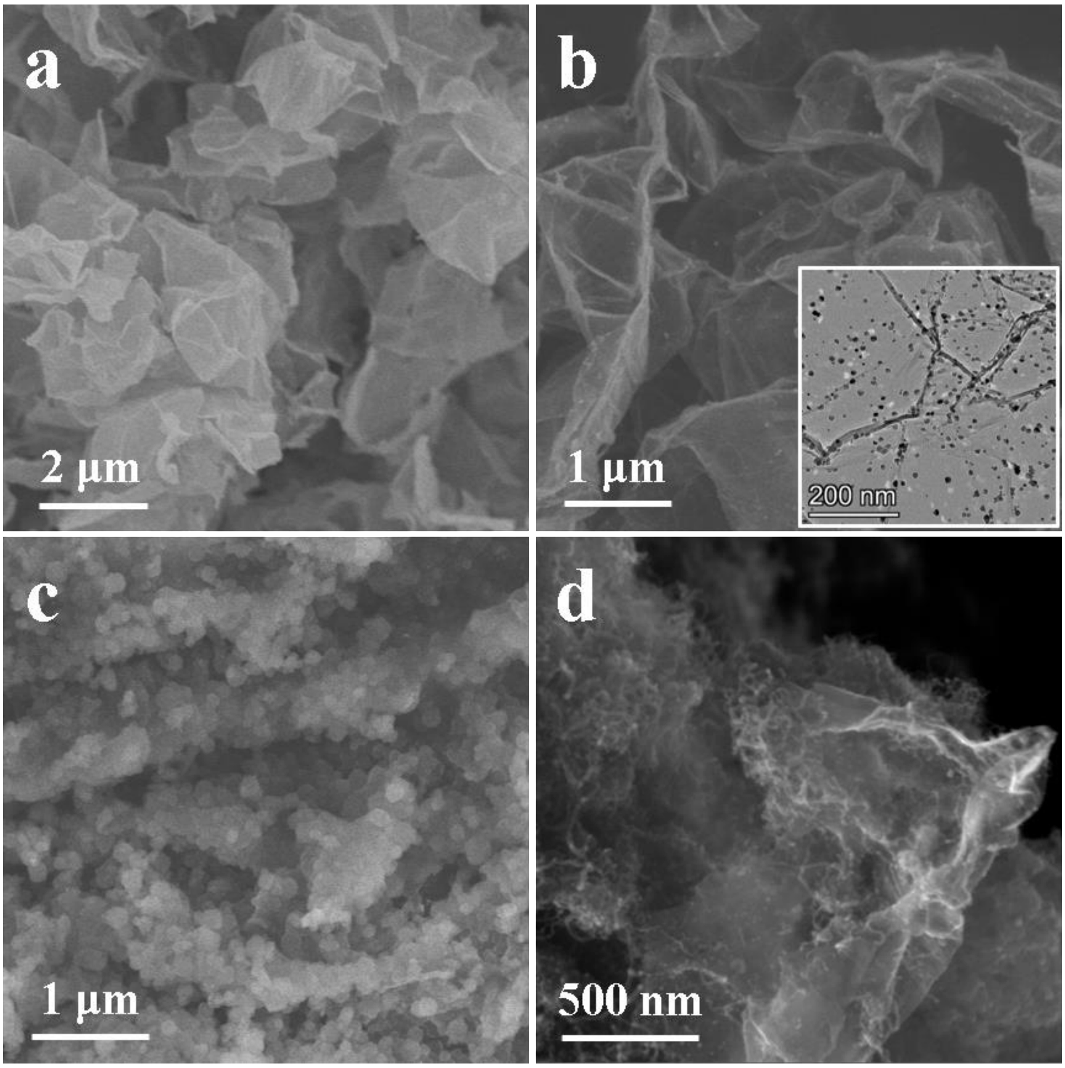

Supplementary Fig. 2 SEM images of (a) rGO, (b) Pt/rGO, (c) Pt@ZIF-67/rGO, and (d) PtCo@CoNC/NTG.

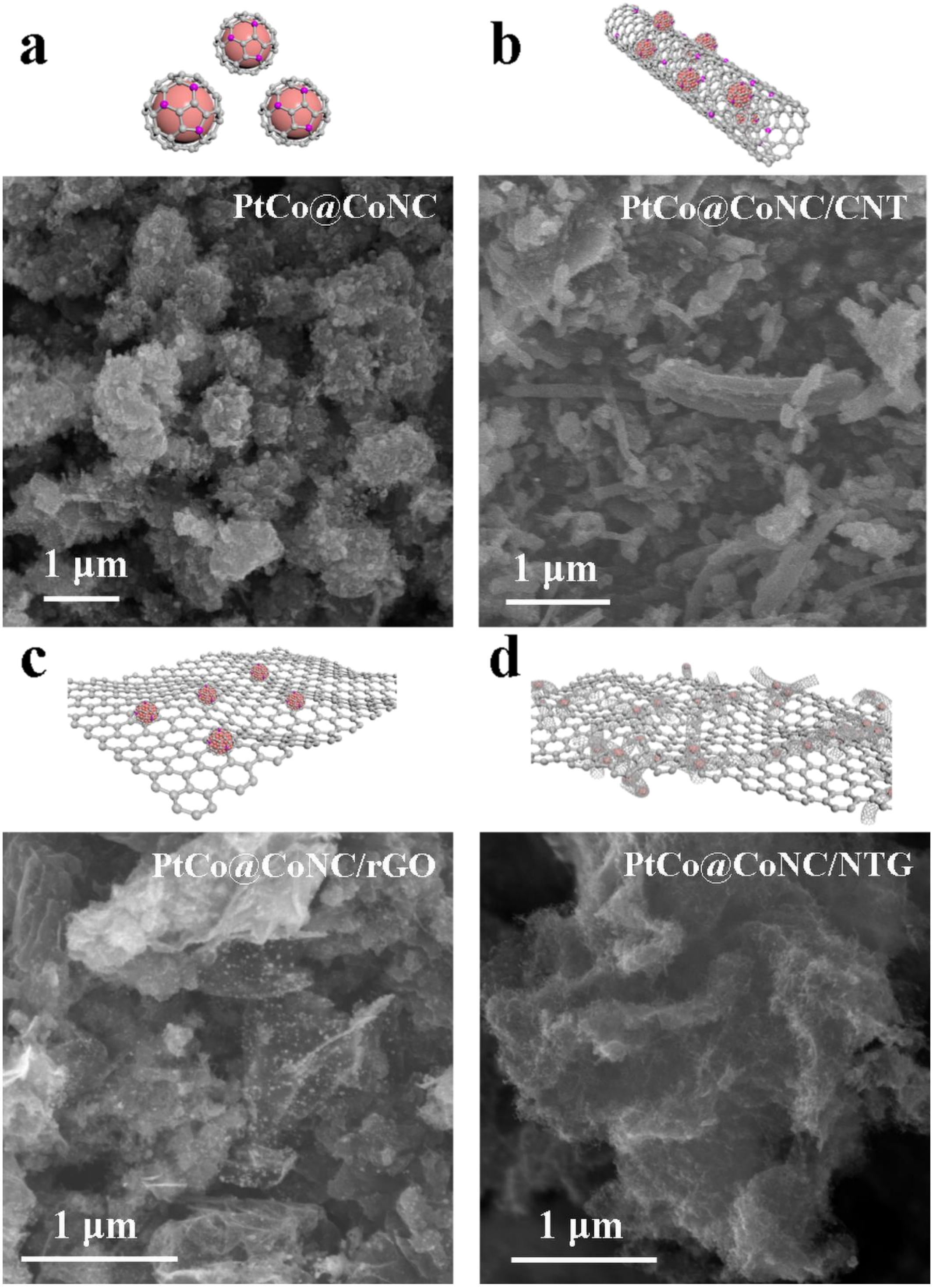

Supplementary Fig. 3 The models and corresponding SEM images of (a) PtCo@CoNC, (b) PtCo@CoNC/CNT, (c) PtCo@CoNC/rGO and (d) PtCo@CoNC/NTG.

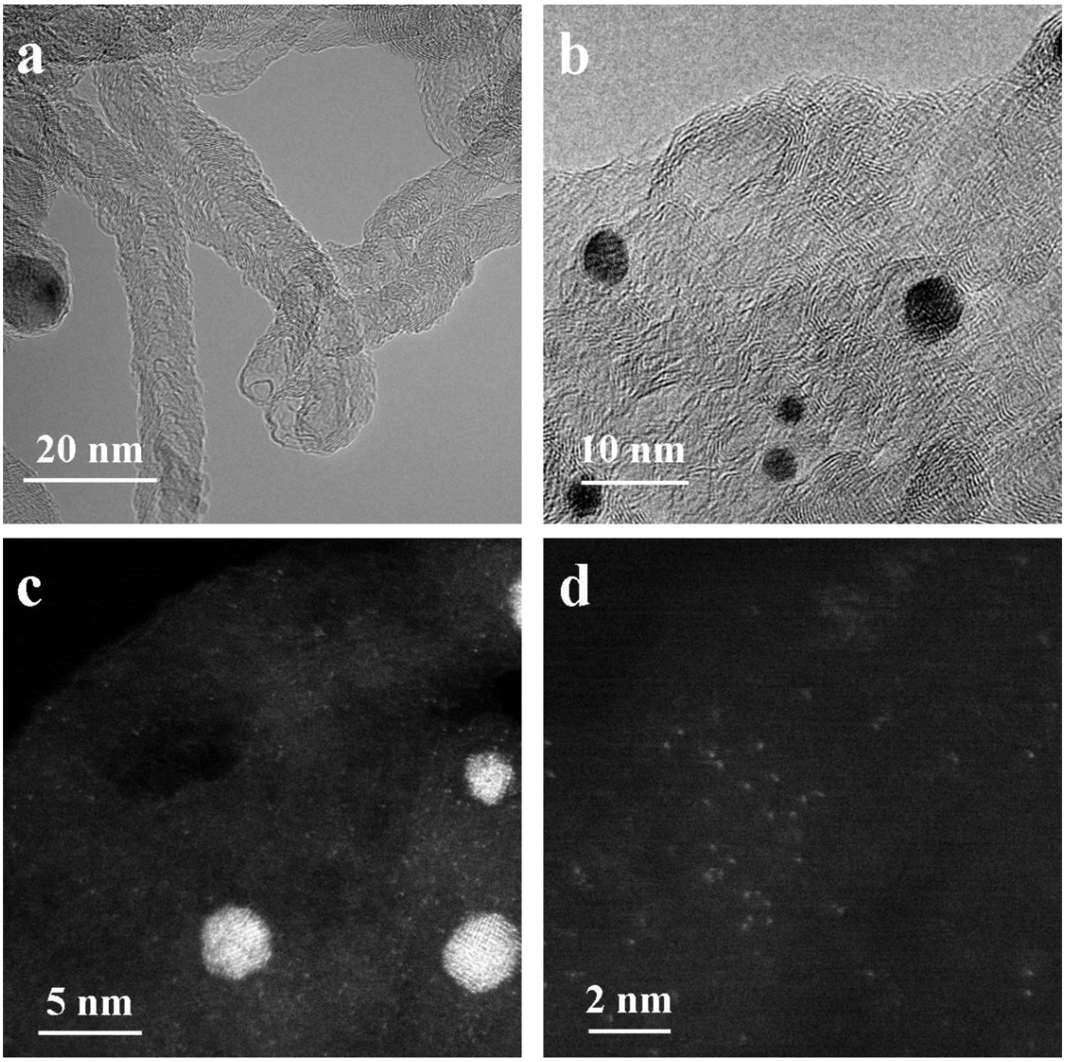

Supplementary Fig. 4 (a, b) HRTEM and (c, d) AC-STEM images of PtCo@CoNC/NTG.

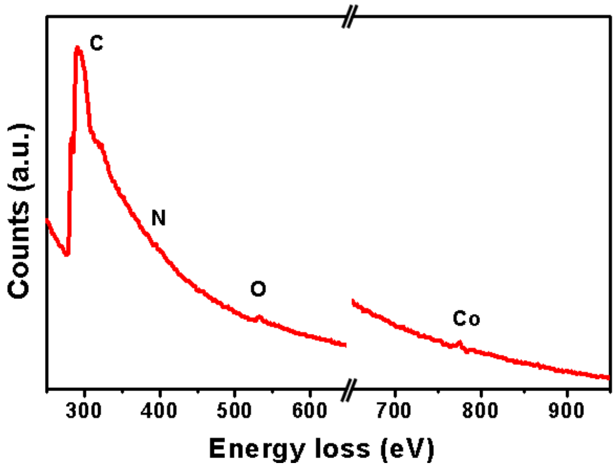

Supplementary Fig. 5 EELS analysis of the elemental composition for the little bright spots.

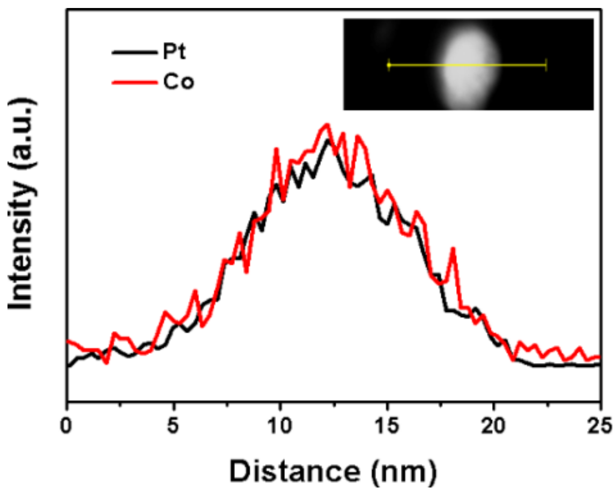

Supplementary Fig. 6 Line scan profile of PtCo particles in PtCo@CoNC/NTG.

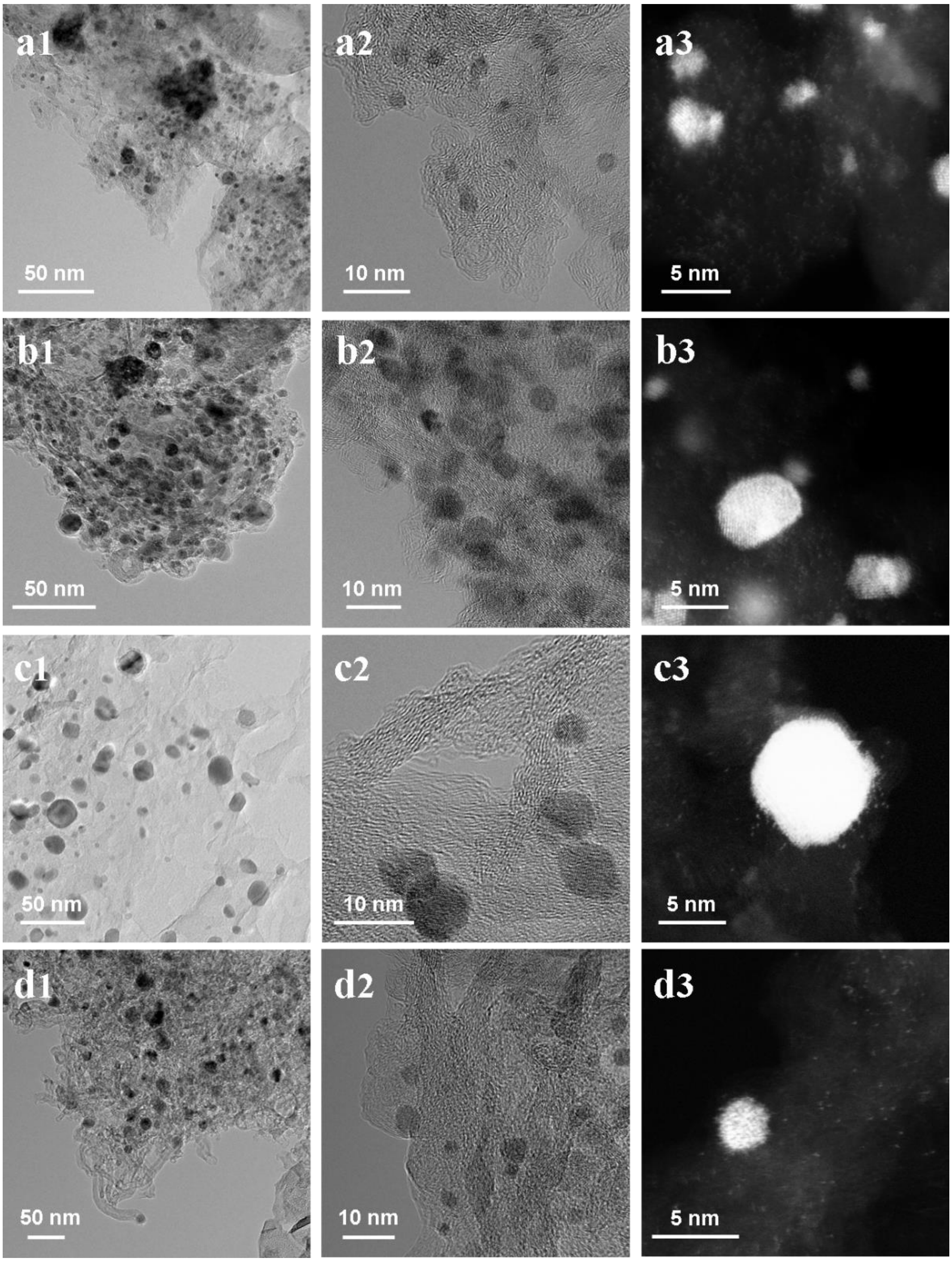

Supplementary Fig. 7 TEM, HRTEM and AC-STEM images of (a) PtCo@CoNC, (b) PtCo@CoNC/CNT, (c) PtCo@CoNC/rGO and (d) PtCo@CoNC/NTG.

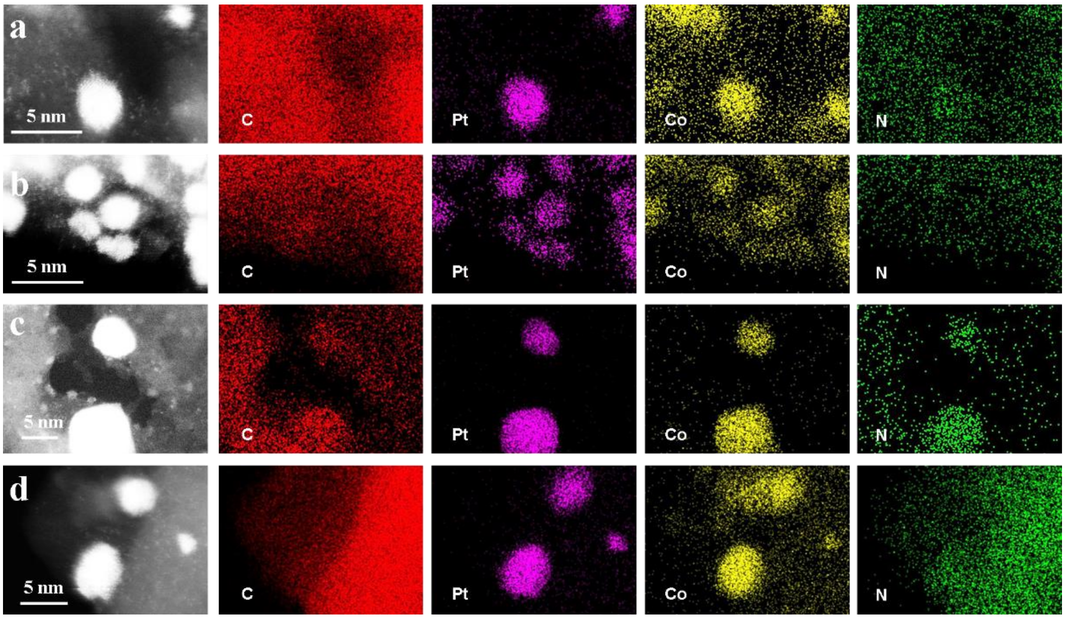

Supplementary Fig. 8 STEM images and corresponding EDS mapping of (a) PtCo@CoNC, (b) PtCo@CoNC/CNT, (c) PtCo@CoNC/rGO and (d) PtCo@CoNC/NTG.

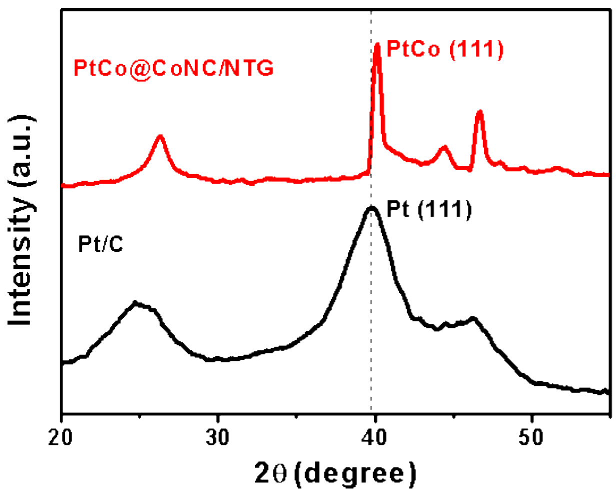

Supplementary Fig. 9 XRD patterns of PtCo@CoNC/NTG and commercial Pt/C.

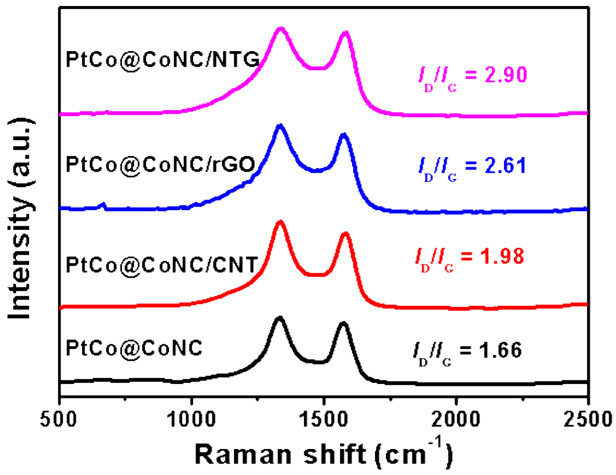

Supplementary Fig. 10 Raman spectra of different PtCo@CoNC samples.

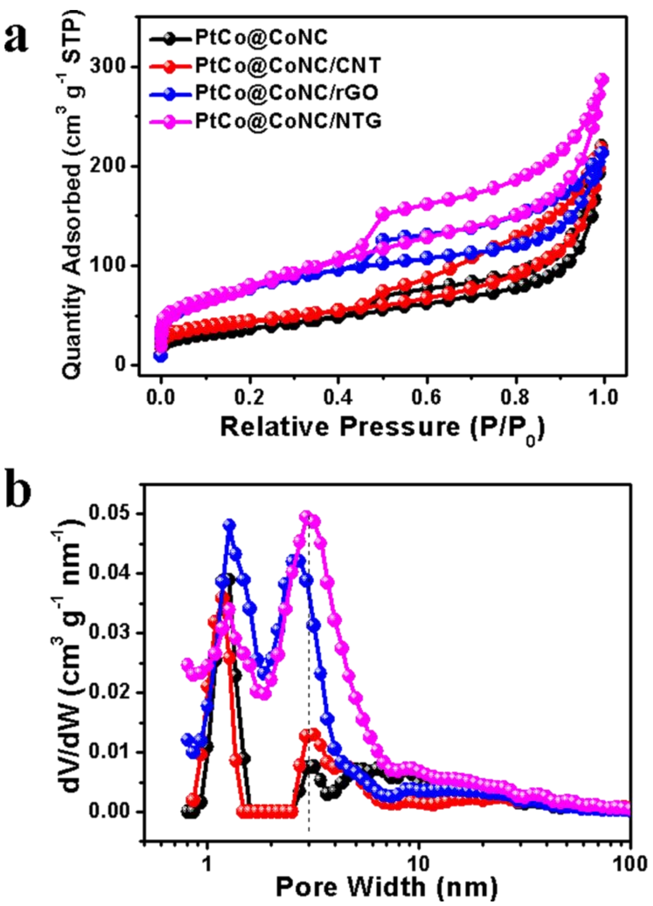

Supplementary Fig. 11 (a) N_2 adsorption and desorption curves and (b) pore width distribution of different PtCo@CoNC samples.

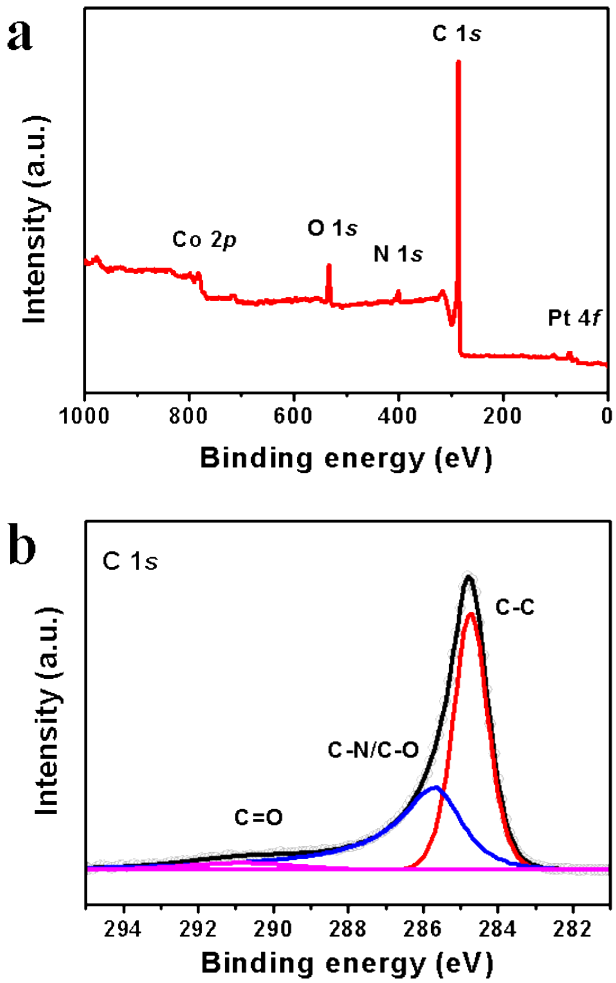

Supplementary Fig. 12 (a) XPS survey, (b) C 1 s signal of PtCo@CoNC/NTG.

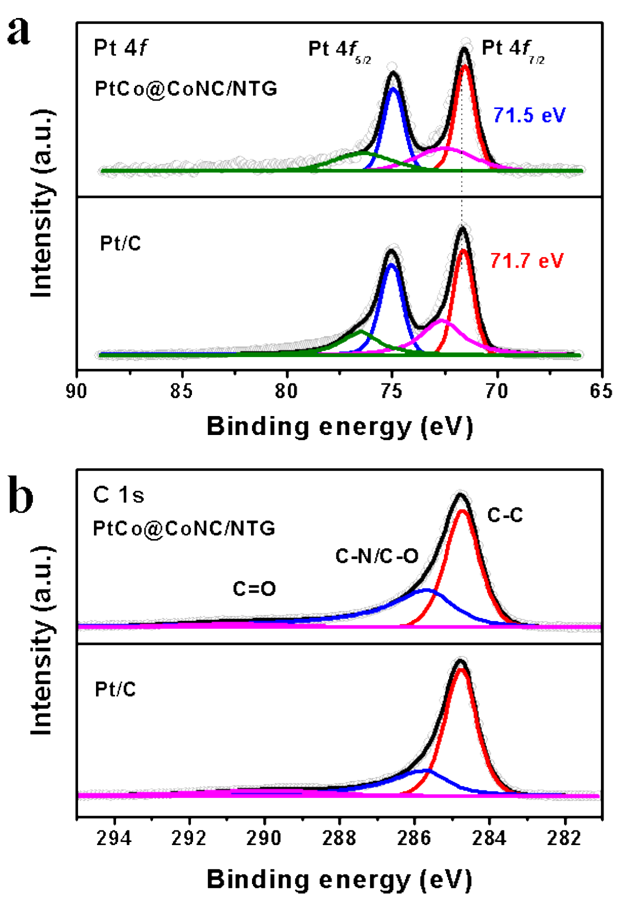

Supplementary Fig. 13 (a) Pt 4f, (b) C 1 s spectra of PtCo@CoNC/NTG and Pt/C.

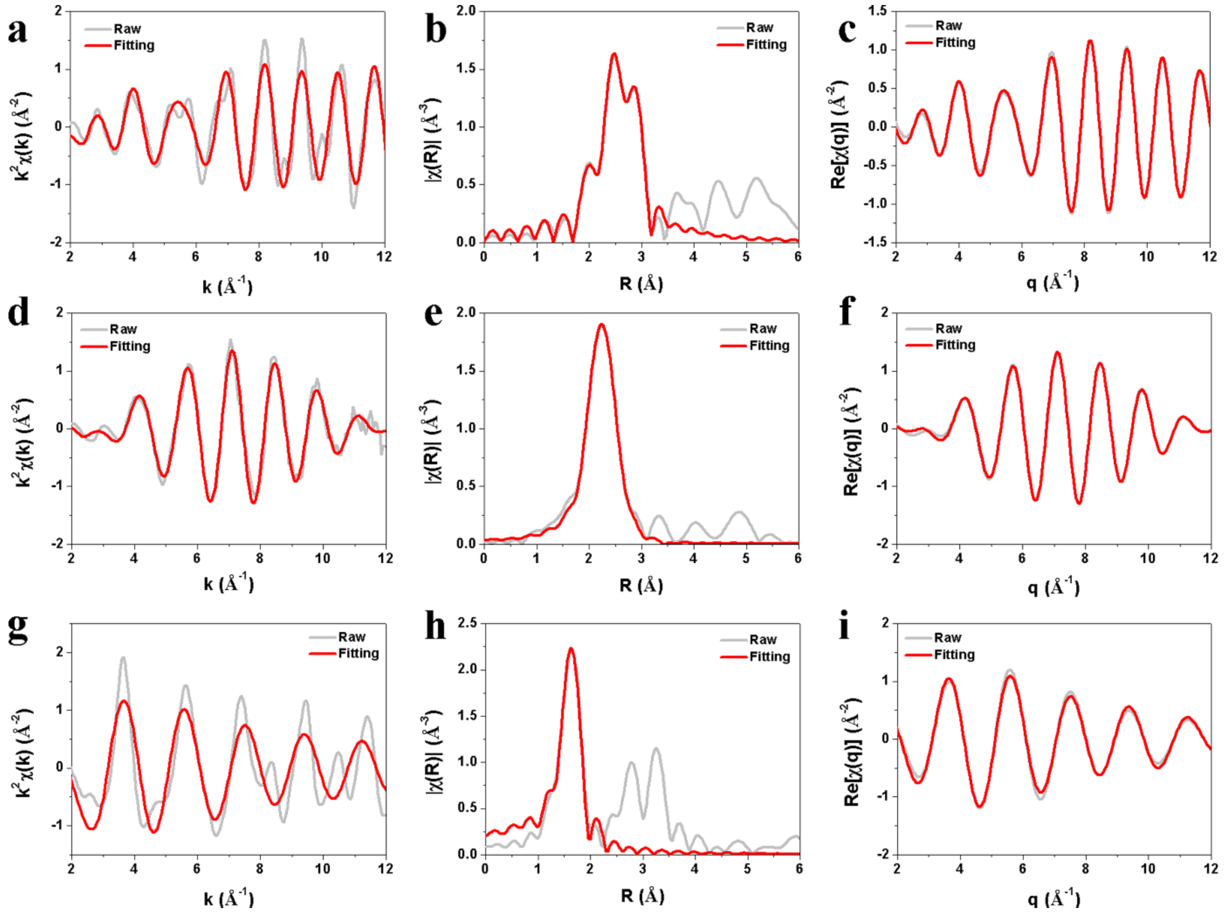

Supplementary Fig. 14 k, R space and inverse FT-EXAFS fitting results of Pt L_3-edge for (a-c) Pt foil (FT range: 2.0-12.0 Å^{-1}; fitting range: 1.1-3.1 Å), (d-f) PtCo@CoNC/NTG (FT range: 2.0-12.0 Å^{-1}; fitting range: 1.0-3.0 Å), and (g-i) PtO_2 (FT range: 2.0-12.0 Å^{-1}; fitting range: 1.1-2.1 Å).

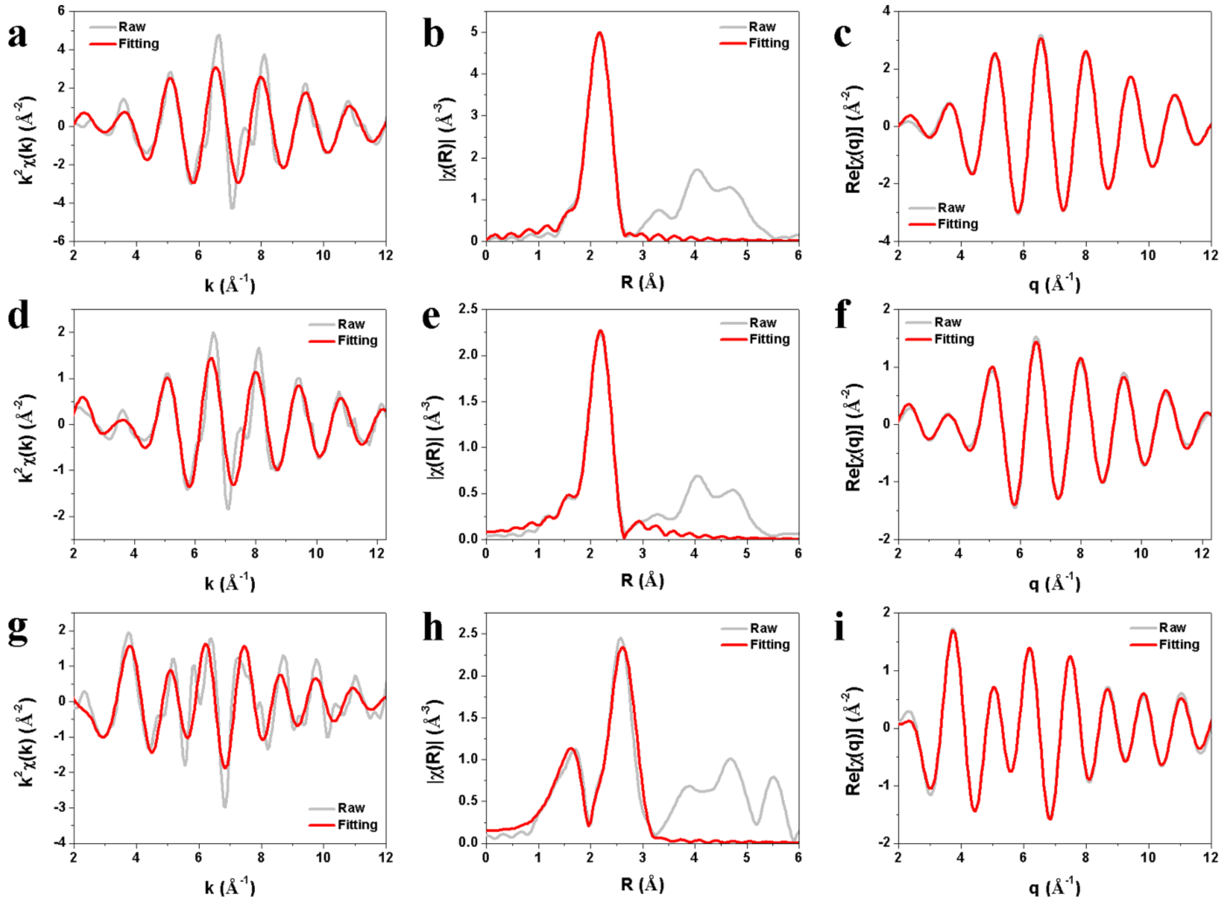

Supplementary Fig. 15 k, R space and inverse FT-EXAFS fitting results of Co K-edge for (a-c) Co foil (FT range: 2.0-12.0 $Å^{-1}$; fitting range: 1.0-3.0 Å), (d-e) PtCo@CoNC/NTG (FT range: 2.0-12.3 $Å^{-1}$; fitting range: 1.0-3.3 Å), and (g-i) CoO (FT range: 2.0-12.0 $Å^{-1}$; fitting range: 1.1-2.8 Å).

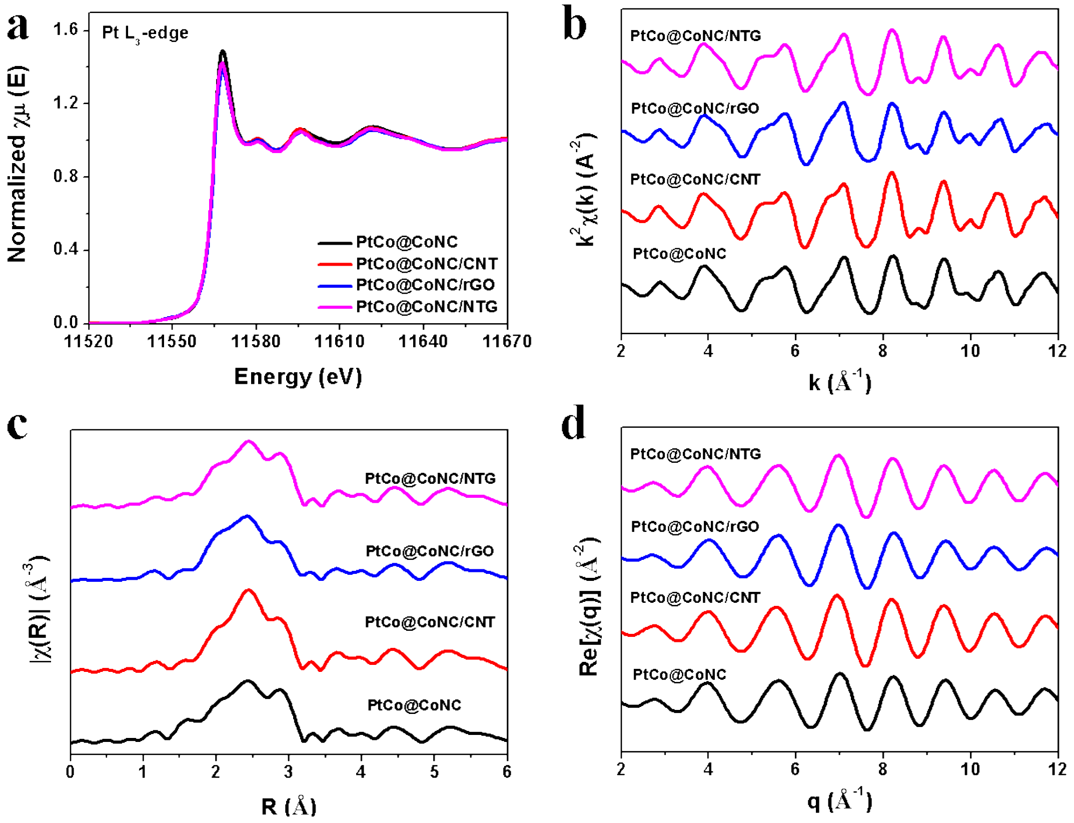

Supplementary Fig. 16 (a) Pt L_3-edge XANES, (b) k space, (c) R space, and (d) inverse FT-EXAFS for PtCo@CoNC hybrids (k-weight = 2; k range = 2.0-12.0 Å^{-1}).

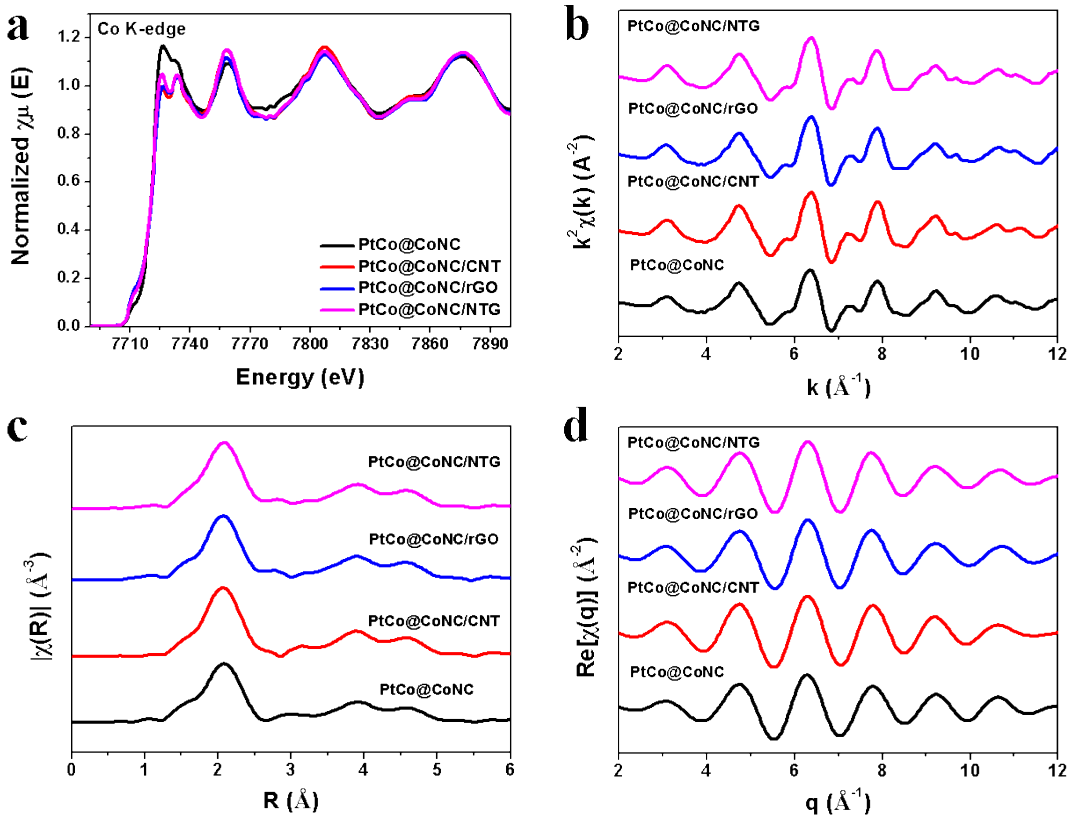

Supplementary Fig. 17 (a) Co K-edge XANES, (b) k space, (c) R space, and (d) inverse FT-EXAFS for PtCo@CoNC hybrids (k-weight = 2; k range = 2.0-12.0 Å^{-1}).

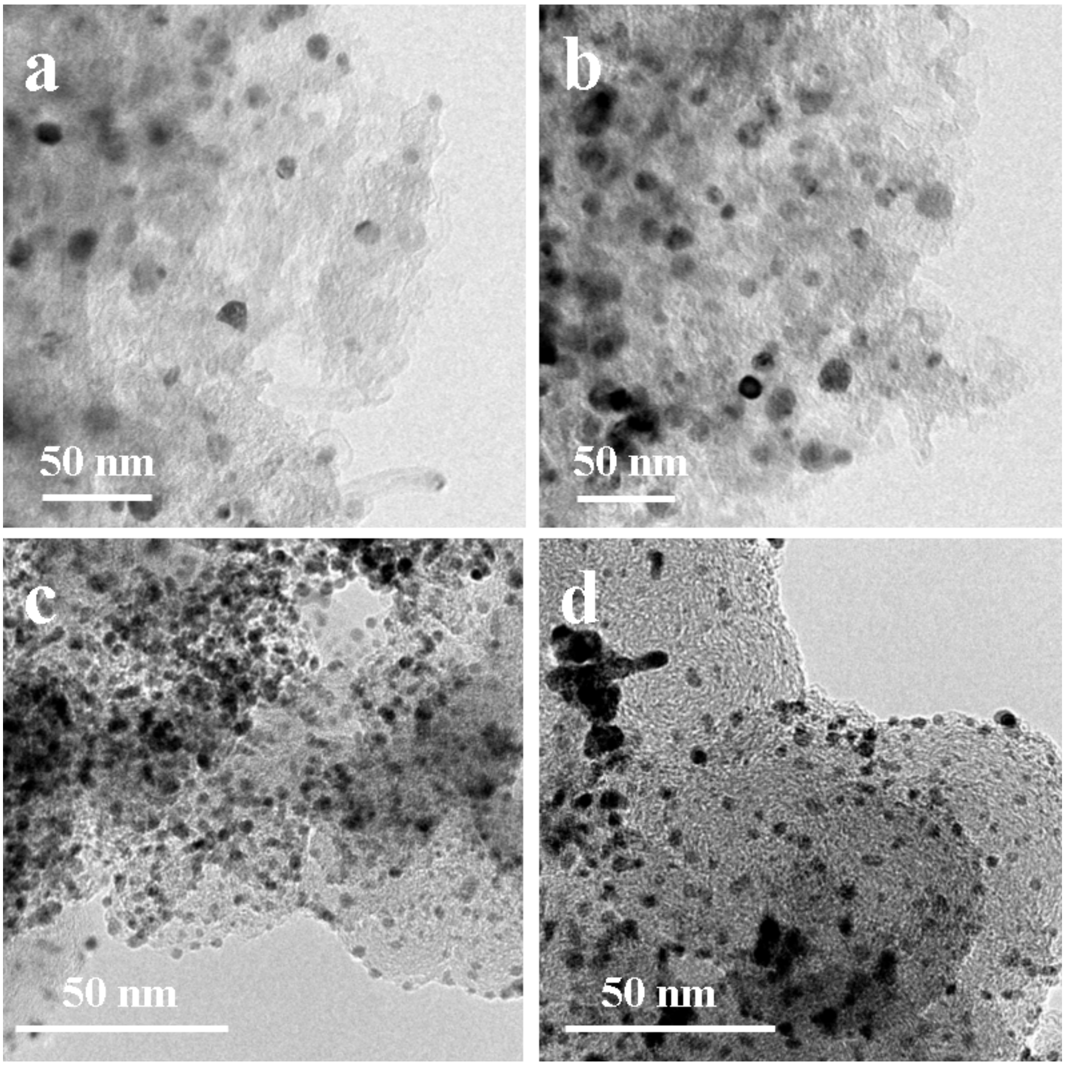

Supplementary Fig. 18 TEM images of (a, b) PtCo@CoNC/NTG and (c, d) commercial Pt/C before and after ADTs.

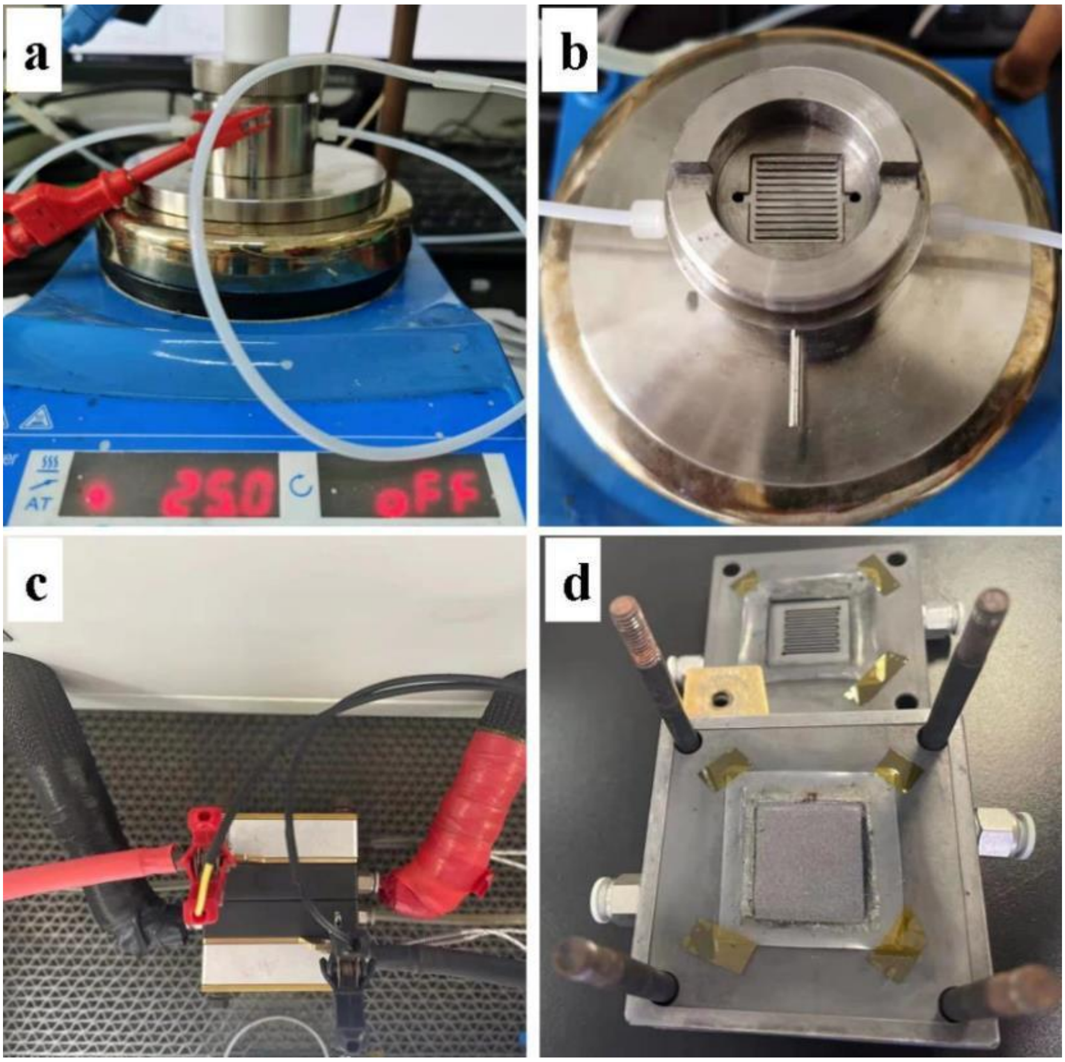

Supplementary Fig. 19 (a, b) GDE evaluation (electrode area: 0.502 cm^2) and (c, d) single hydrogen-air fuel cell configurations (electrode area: 4.0 cm^2).

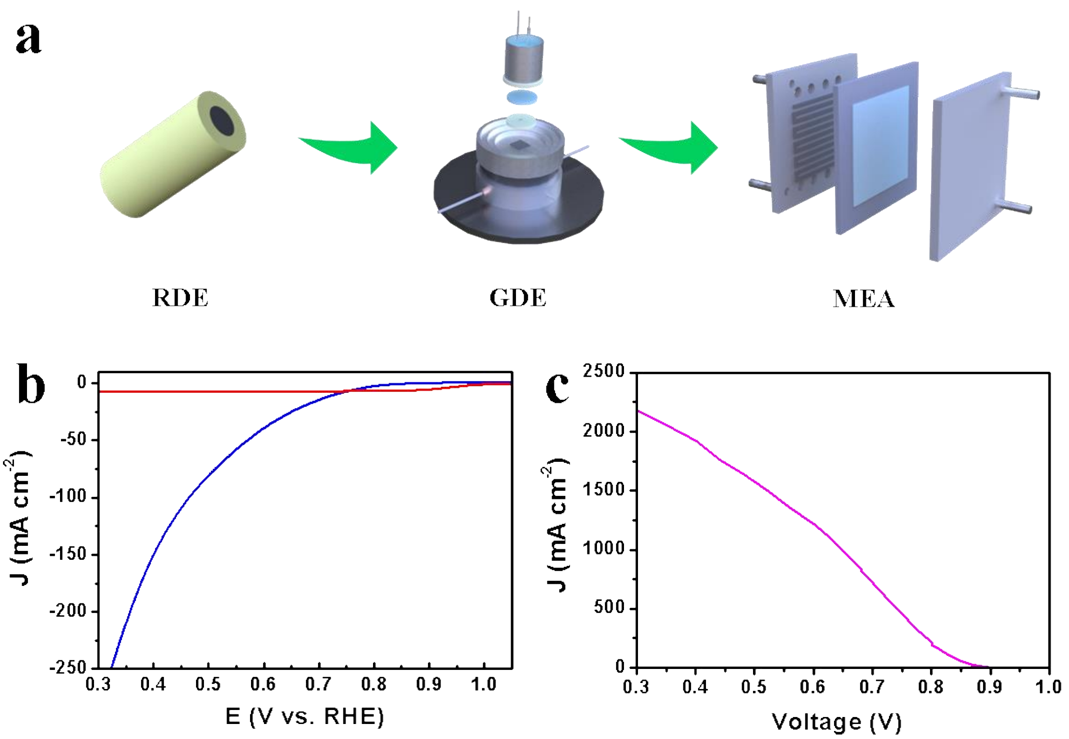

Supplementary Fig. 20 (a) Multilevel electrocatalytic ORR evaluations. ORR polarization curves evaluated by (b) RDE and GDE and (c) MEA for the PtCo@CoNC/NTG catalyst.

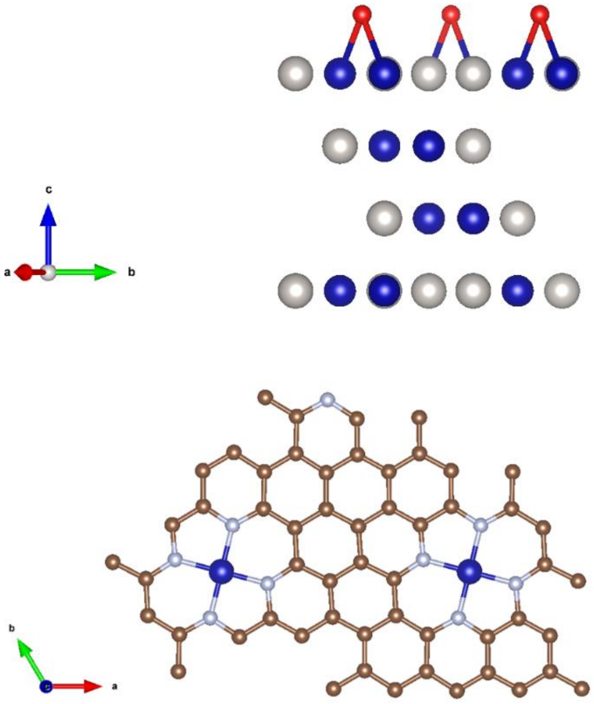

Supplementary Fig. 21 ORR adsorption models of PtCo and Co-N-C for DFT calculations.

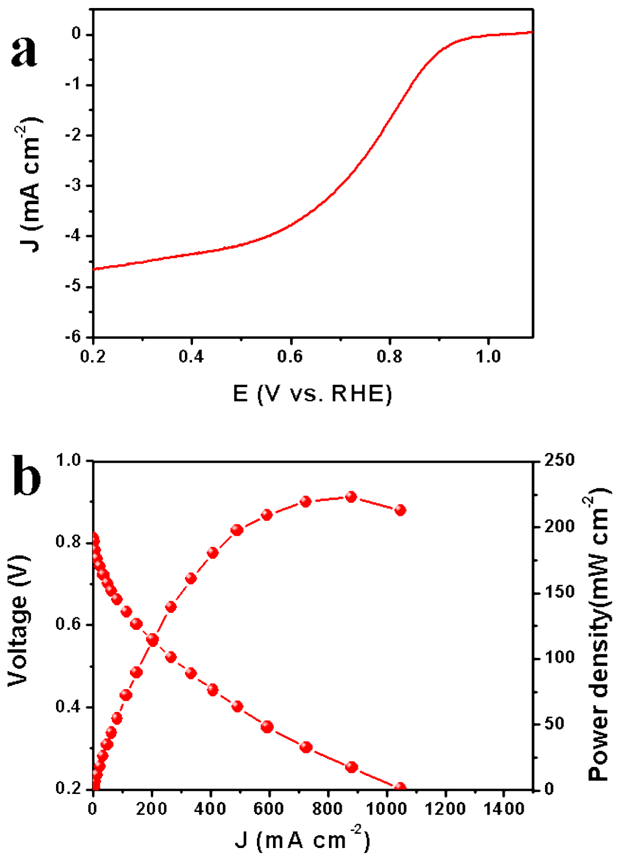

Supplementary Fig. 22 ORR performance evaluated by (a) RDE and (b) MEA for the Co-N-C catalyst.

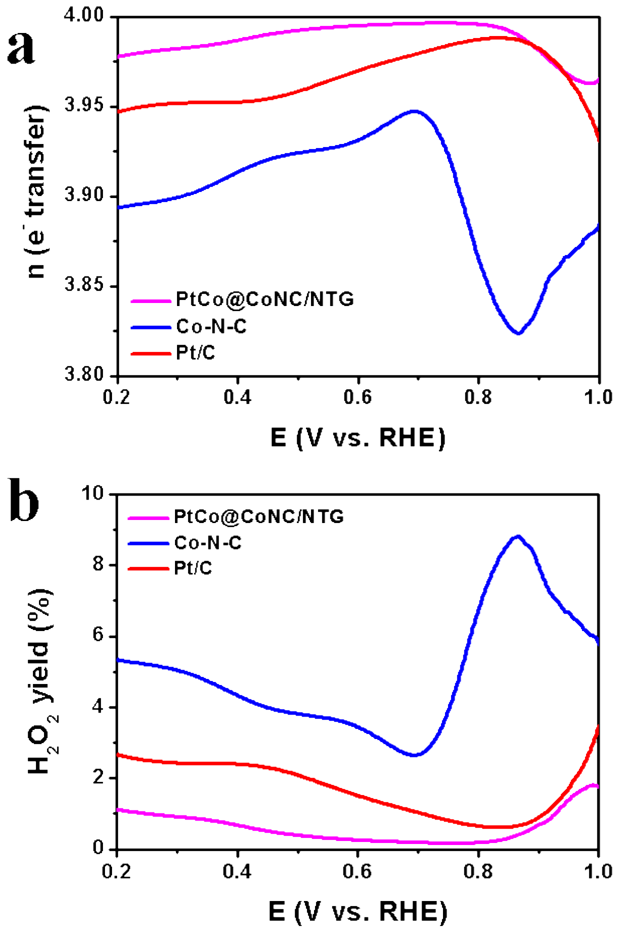

Supplementary Fig. 23 (a) Number of electrons transferred, and (b) H_2O_2 yield by RRDE evaluation for PtCo@CoNC/NTG, Co-N-C and Pt/C catalysts.

Supplementary Table 1. Structural parameters of Pt foil, PtCo@CoNC/NTG, and PtO_2 extracted from the EXAFS fitting (S_0^2= 0.73).

**Sample**	**Bond**	**CN**	**R/Å**	**ΔE_0 /eV**	**σ^2/Å^2**	**R-factor**
Pt foil	Pt-Pt	12*	2.762 ± 0.003	7.242 ± 0.477	0.0040 ± 0.0003	0.004
PtCo@CoNC/ NTG	Pt-Pt	3.74 ± 1.48	2.691 ± 0.010	5.267 ± 0.864	0.0048 ± 0.0021	0.003
	Pt-Co	5.92 ± 0.70	2.578 ± 0.005		0.0057 ± 0.0008	
PtO_2	Pt-O	4.84 ± 0.68	2.018 ± 0.011	12.668± 1.587	0.0002 ± 0.0015	0.016

Note: S_0^2 is the amplitude reduction factor (obtained by the fitting of Pt foil); CN is the coordination number; R is interatomic distance (the bond length between Pt central atoms and surrounding coordination atoms); ΔE_0 is energy shift; σ^2 is Debye-Waller factor (a measure of thermal and static disorder in absorber-scatterer distances); R-factor is used to value the goodness of the fitting; * the experimental EXAFS fit of metal foil by fixing CN as the known crystallographic value.

For the Pt L_3-edge of PtCo@CoNC/NTG, the data ranges were $2.0 < k < 12.0$ Å^{-1} and $1.0 < R < 3.0$ Å. The number of variable parameters was 7 out of a total of 12.44 independent points. The Pt-Pt is based on the crystal structure of PtCo.

Supplementary Table 2. Structural parameters of Co foil, PtCo@CoNC/NTG, and CoO extracted from the EXAFS fitting (S_0^2= 0.84).

**Sample**	**Bond**	**CN**	**R/Å**	**ΔE_0 /eV**	**σ^2/Å^2**	**R-factor**
Co foil	Co-Co	12*	2.489 ± 0.003	6.296 ± 0.493	0.0068 ± 0.0004	0.004
PtCo@CoNC/ NTG	Co-Co	5.25 ± 0.71	2.498 ± 0.007	7.706 ± 1.343	0.0062 ± 0.0010	0.009
	Co-N	0.77 ± 0.52	2.003 ± 0.044		0.0069 ± 0.0126	
	Co-Pt	1.80 ± 1.39	2.813 ± 0.047		0.0121 ± 0.0085	
CoO	Co-O	4.76 ± 0.95	2.119 ±0.016	1.356 ± 1.164	0.0080 ± 0.0034	0.010
	Co-Co	14.90 ± 2.80	3.033 ± 0.011		0.0101 ± 0.0018	

Note: S_0^2 is the amplitude reduction factor (obtained by the fitting of Co foil); CN is the coordination number; R is interatomic distance (the bond length between Co central atoms and surrounding coordination atoms); ΔE_0 is energy shift; σ^2 is Debye-Waller factor (a measure of thermal and static disorder in absorber-scatterer distances); R-factor is used to value the goodness of the fitting; * the experimental EXAFS fit of metal foil by fixing CN as the known crystallographic value.

For Co K-edge of PtCo@CoNC/NTG, the data range was $2.0 < k < 12.3$ Å^{-1} and $1.0 < R < 3.3$ Å. The number of variable parameters was 10 out of 14.81 independent points. The distances of Co-N and CoPt are based on the crystal structures of CoN and PtCo.

Supplementary Table 3. Electrochemical evaluations of commercial Pt/C and different PtCo@CoNC catalysts.

Catalyst	Pt mass ratio (wt%)	RDE			GDE			MEA			
		Pt loading (μg cm^{-2})	Half-wave potential (V)	Mass activity$_{0.9}$ (A mg$_{Pt}^{-1}$)	Pt loading (mg$_{Pt}$ cm^{-2})	Current density$_{0.6}$ (mA cm^{-2})	Mass activity$_{0.6}$ (A mg$_{Pt}^{-1}$)	Anode loading (mg$_{Pt}$ cm^{-2})	Cathode loading (mg$_{Pt}$ cm^{-2})	Current density$_{0.6}$ (A cm^{-2})	Power density (mW cm^{-2})
Pt/C	40.0	20.4	0.87	0.13	0.04	30.2	0.75	0.1	0.2	1.21	780
PtCo@CoNC	3.2	8.1	0.86	0.25	0.02	9.4	0.47	0.1	0.1	0.84	680
PtCo@CoNC/CNT	2.9	7.4	0.88	0.42	0.02	16.1	0.81	0.1	0.1	1.03	820
PtCo@CoNC/rGO	2.5	6.3	0.89	0.85	0.02	17.5	0.88	0.1	0.1	1.04	850
PtCo@CoNC/NTG	4.5	11.5	0.94	1.52	0.02	38.9	1.95	0.1	0.1	1.50	980

Supplementary Table 4. Comparison of our PtCo@CoNC, commercial Pt/C and some carbon-supported ORR electrocatalysts in hydrogen-air fuel cells.

Catalysts	Anode Pt loading ($mg\ cm^{-2}$)	Cathode Pt loading ($mg\ cm^{-2}$)	Current density at 0.6 V ($mA\ cm^{-2}$)	Power density ($mW\ cm^{-2}$)	Back pressure (MPa)	References
PtCo@CoNC/NTG	0.1	0.1	1.50	980	0.2	This work
PtCo@CoNC/rGO	0.1	0.1	1.04	850		
PtCo@CoNC/CNT	0.1	0.1	1.03	820		
PtCo@CoNC	0.1	0.1	0.84	680		
Pt/C	0.1	0.2	1.21	780		
JM Pt/C	0.1	0.3	1.0 #	664	H_2, 0.1; Air, 0.2	5
PtNi/C	0.15	0.15	0.64	490 #	without	6
Ga-PtNi/C	0.15	0.15	0.70	540 #		
PtNi-BNSs	0.1	0.15	1.0	700	0.2	7
PtNi-BNCs	0.1	0.15	1.5	920		
Pt/40Co-NC-900	N/A	0.13	1.1 #	700 #	H_2, N/A; Air, 0.1	8
$L1_0$-PtZn	0.1	0.104	1.3 #	800 #	0.15	9
Pt-9.3@NPC 1atm	0.1	0.1	1.1	947	0.1	10
Pt-9.3@NPC 2atm	0.1	0.05	1.2	1071	0.2	
Pt/FeN_4-C	0.1	0.1	1.4	679	0.15	11
Pt_3Co/FeN_4-C	0.1	0.1	1.6	822		
Coplanar Pt/C NMs	0.11	0.1	0.76 #	553	0.15	12
LP@PF1	0.35	0.033	1.45 #	1000	0.2	13
LP@PF2	0.35	0.035	1.45 #	1100		

The data are not given, but excavated from the hydrogen-air fuel cell polarization curves.

Supplementary References

1. Ravel, B., Newville, M. ATHENA, ARTEMIS, HEPHAESTUS: data analysis for X-ray absorption spectroscopy using IFEFFIT. *J. Synchrotron Radiat.* **2005**, *12*, 537.

2. Perdew, J. P., Burke, K., Ernzerhof, M. Generalized gradient approximation made simple. *Phys. Rev. Lett.* **1996**, *77*, 3865.

3. Kresse, G., Joubert, D. From ultrasoft pseudopotentials to the projector augmented-wave method. *Phys. Rev. B* **1999**, *59*, 1758.

4. Nørskov, J. K., Rossmeisl, J., Logadottir, A., Lindqvist, L., Kitchin, J. R., Bligaard, T., Jonsson, H. Origin of the overpotential for oxygen reduction at a fuel-cell cathode. *J. Phys. Chem. B* **2004**, *108*, 17886.

5. Wang, J., Huang, Z., Liu, W., Chang, C., Tang, H., Li, Z., Chen, W., Jia, C., Yao, T., Wei, S., Wu, Y., Li, Y. Design of N-coordinated dual-metal sites: A stable and active Pt-free catalyst for acidic oxygen reduction reaction. *J. Am. Chem. Soc.* 2017*, 139,* 17281.

6. Lim, J., Shin, H., Kim, M., Lee, H., Lee, K. S., Kwon, Y., Song, D., Oh, S., Kim, H., Cho, E. Ga-doped Pt-Ni octahedral nanoparticles as a highly active and durable electrocatalyst for oxygen reduction reaction. *Nano Lett.* 2018*, 18,* 2450.

7. Tian, X., Zhao, X., Su, Y. Q., Wang, L., Wang, H., Dang, D., Chi, B., Liu, H., Hensen, E. J. M. X., Lou, X. W. D., Xia, B. Y. Engineering bunched Pt-Ni alloy nanocages for efficient oxygen reduction in practical fuel cells. *Science* 2019*, 366,* 850.

8. Wang, X. X., Hwang, S., Pan, Y., Chen, K., He, Y., Karakalos, S., Zhang, H., Spendelow, J. S., Su, D., Wu, G. Ordered Pt_3Co intermetallic nanoparticles derived from metal-organic frameworks for oxygen reduction. *Nano Lett.* 2018*, 18,* 4163.

9. Liang, J., Zhao, Z., Li, N., Wang, X., Li, S., Liu, X., Wang, T., Lu, G., Wang, D., Hwang, B., Huang, Y., Su, D., Li, Q. Biaxial strains mediated oxygen reduction electrocatalysis on fenton reaction resistant $L1_0$-PtZn fuel cell cathode. *Adv. Energy Mater.* 2020*, 10,* 2000179.

10. Zhu, S., Wang, X., Luo, E., Yang, L., Chu, Y., Gao, L., Jin, Z., Liu, C., Ge, J., Xing, W. Stabilized Pt cluster-based catalysts used as low-loading cathode in proton-exchange membrane fuel cells. *ACS Energy Lett.* 2020*, 5,* 3021.

11. Qiao, Z., Wang, C. Y., Li, C. Z., Zeng, Y. C., Hwang, S., Li, B. Y., Karakalos, S., Park, J., Kropf, A. J., Wegener, E. C., Gong, Q., Xu, H., Wang, G. F., Myers, D. J., Xie, J., Spendelow, J. S., Wu, G. Atomically dispersed single iron sites for promoting Pt and Pt_3Co fuel cell catalysts: performance and durability improvements. *Energy Environ. Sci.* 2021, *14*, 4948.

12. Hu, Y., Zhu, M., Luo, X., Wu, G., Chao, T., Qu, Y., Zhou, F., Sun, R., Han, X., Li, H., Jiang, B., Wu, Y., Hong, X. Coplanar Pt/C nanomeshes with ultrastable oxygen reduction performance in fuel cells. *Angew. Chem. Int. Ed.* 2021, *60,* 6533.

13. Chong, L., Wen, J., Kubal, J., Sen, F. G., Zou, J., Greeley, J., Chan, M., Barkholtz, H., Ding, W., Liu, D. J. Ultralow-loading platinum-cobalt fuel cell catalysts derived from imidazolate frameworks. *Science* 2018*, 362,* 1276.
